# Mitochondrial disease: genetics and management

**DOI:** 10.1007/s00415-015-7884-3

**Published:** 2015-08-28

**Authors:** Yi Shiau Ng, Doug M. Turnbull

**Affiliations:** Wellcome Trust Centre for Mitochondrial Research, Institute of Neuroscience, The Medical School, Newcastle University, Framlington Place, Newcastle upon Tyne, NE2 4HH UK

**Keywords:** Mitochondrial disease, Mitochondrial DNA (mtDNA), Nuclear genes, Acute and chronic neurological presentations, Treatment

## Abstract

Mitochondrial disease is one of the most common groups of genetic diseases with a minimum prevalence of greater than 1 in 5000 in adults. Whilst multi-system involvement is often evident, neurological manifestation is the principal presentation in most cases. The multiple clinical phenotypes and the involvement of both the mitochondrial and nuclear genome make mitochondrial disease particularly challenging for the clinician. In this review article we cover mitochondrial genetics and common neurological presentations associated with adult mitochondrial disease. In addition, specific and supportive treatments are discussed.

## Introduction

Mitochondrial disease is a collective term that encompasses the genetically and clinically heterogeneous group of diseases due to defects in mitochondrial oxidative phosphorylation. It is one of the most common groups of genetic disease and can be caused by mutation in either mitochondrial DNA or nuclear genes that directly or indirectly interfere with the mitochondrial respiratory chain function. To date, mitochondrial proteomics analysis reveals that in addition to the 13 proteins encoded by the mitochondrial genome, around 1500 proteins [50] are linked to various mitochondrial functions and so far more than 200 genes have been implicated in the development of human disease [40].

A number of syndromes have been described in mitochondrial disease but often patients present with non-syndromic presentation of which nervous system is most commonly affected [49]. In addition to the diagnostic challenge, clinicians also encounter difficulty in the management of mitochondrial disease due to lacking of effective disease-modifying therapy and, until recently, best practice guidelines on various complications associated with the disease [59].

In this review article, we discuss the genetics and epidemiology of mitochondrial disease, neurological presentations and their management, genetic counselling and reproductive options for patients.

## Mitochondrial genetics

Mitochondria are cellular organelles found in all nucleated human cells. A crucial function of mitochondria is to generate energy in the form of ATP (adenosine triphosphate) via oxidative phosphorylation using predominantly carbohydrates and fatty acids as fuel. The oxidative phosphorylation system (OXPHOS) is located in the inner membrane and it consists of five multimeric protein complexes: complex I-IV form the respiratory chain and complex V (ATP synthase). In addition, there are two mobile electron carriers (co-enzyme Q10 and cytochrome *c*).

Mitochondria are under dual genetic control of the mitochondrial and nuclear genomes. The mitochondrial genome consists of multiple copies of 16,569 bp, double stranded mitochondrial DNA (mtDNA) molecules and located adjacent to the OXPHOS system in the matrix. Only thirty-seven genes (22 transfer RNAs, 2 ribosomal RNAs and 13 polypeptides that form structural subunits of OXPHOS system) [86] are encoded by mtDNA. The remaining mitochondrial proteins, including the majority of respiratory chain subunits (79 out of 92), assembly factors of the respiratory chain, those involved in maintenance and expression of mtDNA, mtDNA transcription and translation, and control the mitochondrial dynamics are nuclear encoded [16], synthesised in the cytosol and imported to the mitochondria [51].

There are several unique properties associated with the mitochondrial genome that are important in understanding the primary mitochondrial DNA disease: (1) there are multiple copies (up to thousands) of mtDNA in each cell; (2) mtDNA is maternally inherited; (3) the phenomenon of homoplasmy and heteroplasmy. Homoplasmy implies all mtDNA are identical which could be all wild type or mutated. Heteroplasmy is a mixture of mutated and wild type mtDNA. In the presence of heteroplasmy there is a threshold effect and clinical expression can vary between different tissues and mtDNA mutations. In women with heteroplasmic mtDNA mutations there is a bottleneck in the female germline which means that the transmission of heteroplasmy level from mother to offspring is often random and unpredictable. This explains the heterogeneity in heteroplasmy level, clinical phenotype and severity frequently observed within the same pedigree.

Multiple mtDNA deletions and mtDNA depletion (reduced copy number of mtDNA) are secondary changes in mtDNA due to mutations in the mtDNA replication and/or maintenance genes such as *POLG, PEO1, ANT1, DGUOK, TYMP* [72]. Mitochondrial depletion syndrome is associated with infantile/early childhood onset, multi-system disease with fatal outcome and multiple deletions generally result in later onset (child- or adulthood) and milder disease burden.

The emergence of next generation sequencing is leading to rapid discovery of new nuclear genes linked to mitochondrial disease and the classification of nuclear gene related mitochondrial disease is summarised in Table 1. There is also increased recognition of mitochondrial dysfunction and genetic mutation in various other genetic neurological disorders, for examples *SPG7* gene in hereditary spastic paraplegia [58] and ataxia [61] and *GDAP1* gene in Charcot-Marie-Tooth disease type 4A [56, 96].

**Table 1:** Classification of nuclear gene related mitochondrial disease based on the gene function [1, 5, 16, 20, 71, 76, 94]

	Gene	Inheritance	Onset	Common clinical manifestation/syndrome
OXPHOS system				
Complex I				
Structural subunit	*NDUFA2, NDUFA9, NDUFA10, NDUFA11, NDUFA12, NDUFA13, NDUFB3, NDUFB9, NDUFS1, NDUFS2, NDUFS3, NDUFS4, NDUFS6, NDUFS7, NDUFS8*	AR	Infancy	Leigh syndrome, encephalopathy, cardiomyopathy, epilepsy, lactic acidosis
	*NDUFA1, NDUFB11*	X-linked		Leigh syndrome, microphthalmia with linear skin defects
Assembly factor	*ACAD9, NDUFAF1, NDUFAF2, NDUFAF4, NDUFAF5, NDUFAF6, FOXRED1*	AR		Leigh syndrome, encephalopathy, cardiomyopathy
Complex II				
Structural subunit	*SDHA*-*D*	AR	Infancy, childhood	Leigh syndrome, dilated cardiomyopathy
		AD	Childhood, adulthood	Hereditary paraganglioma, pheochromocytoma
Assembly factor	*SDHAF1*	AR	Infancy	Spastic quadriplegia, leukodystrophy
	*SDHAF2*	AD	Adulthood	Hereditary paraganglioma, pheochromocytoma
Complex III				
Structural subunit	*UQCRB*	AR	Childhood	Hypoglycaemia, lactic acidosis
	*UQCRQ*	AR	Childhood	Extrapyramidal signs, ataxia, psychomotor retardation
Assembly factor	*BCS1L*	AR	Infancy	Growth retardation, aminoaciduria, cholestasis, iron overload, and early death (GRACILE)
	*TTC19*	AR	Childhood, adulthood	Encephalomyopathy, ataxia
Complex IV				
Structural subunit	*COX6B1, NDUFA4*	AR	Infancy, childhood	Encephalomyopathy, Leigh-like syndrome
	*COX4I2*	AR	Infancy, childhood	Exocrine pancreatic insufficiency, dyserythropoetic anaemia (similar to Pearson syndrome), calvarial hyperostosis
Assembly factor	*SURF1, SCO1*-*2, COX10, COX15, LRPPRC, COA5, PET100*	AR	Infancy	Leigh syndrome, French-Canadian Leigh syndrome, hypertrophic cardiomyopathy, encephalomyopathy
Complex V				
Structural subunit	*ATP5E*	AR	Infancy	Encephalopathy, dysmorphic features, hypertrophic cardiomyopathy, lactic acidosis
Assembly factor	*ATPAF2, TMEM70*	AR		
Co-enzyme Q10 biosynthesis				
Co-enzyme Q10 deficiency	*COQ2, COQ4, COQ6, PDSS2*	AR	Infancy, childhood	Encephalomyopathy, nephrotic syndrome, sensori-neural deafness
	*ETFDH*	AR	Infancy, childhood, adulthood	Glutaric acidaemia IIC (multiple acyl-CoA dehydrogenase deficiency, MADD), Myopathy
	*ADCK3*	AR	Childhood	Cerebellar ataxia
Mitochondrial DNA replication				
MtDNA depletion or multiple deletions	*POLG*	AD	Adulthood	Chronic progressive external ophthalmoplegia (CPEO)
		AR	Infancy, childhood, adulthood	CPEO, Alpers disease, ataxia-neuropathy syndrome, Leigh syndrome, epilepsy (occipital), Parkinsonism
	*PEO1* (*c10orf2*)	AD	Adulthood	CPEO
		AR	Infancy	Inherited Infancy onset of spinocerebellar ataxia (IOSCA), hepatocerebral syndrome
MtDNA multiple deletions	*POLG2*	AD	Adulthood	CPEO
Nucleotide synthesis and transport (maintenance)				
MtDNA depletion or multiple deletions	*TYMP*	AR	Childhood, adulthood	Mitochondrial neuro-gastrointestinal encephalomyopathy (MNGIE)
	*RRM2B*	AD	Adulthood	CPEO
		AR	Infancy	Encephalomyopathy, gut dysmotility, Kearns-Sayre syndrome (KSS), proximal renal tubulopathy
	*SLC25A4* (*ANT1*)	AD	Adulthood	CPEO
		AR	Childhood	Hypertrophic cardiomyopathy, lactic acidosis
	*TK2*	AR	Infancy, childhood, adulthood	Myopathy and respiratory muscle weakness
	*MPV17*	AR	Infancy, childhood, adulthood	Hepatocerebral syndrome, neuropathy and leukoencephalopathy
MtDNA depletion	*DGUOK*	AR	Infancy	Hepatocerebral syndrome
	*SUCLA2, SUCLG1*	AR	Infancy	Encephalomyopathy, raised methylmalonic acid, hyperkinesia
MtDNA multiple deletions	*DNA2*	AD	Adulthood	CPEO
No change in mtDNA content/unknown	*SLC25A3*	AR	Infancy	Hypertrophic cardiomyopathy, hypotonia
	*GFER*	AR	Childhood, adulthood	Congenital cataract, myopathy, cardiomyopathy
Mitochondrial translation				
Multiple respiratory chain deficiency				
Ribosomal protein	*MRPS16*	AR	Infancy	Dysmorphism, lactic acidosis, agenesis of corpus callosum
	*MRPS22*	AR	Infancy	Cardiomyopathy, tubulopathy, hypotonia
Elongation factor	*GFM1, TUFM*	AR	Infancy	Leigh syndrome
	*TSFM*	AR	Infancy, childhood	Encephalopathy, hypertrophic cardiomyopathy
tRNA modification	*PUS1*	AR	Infancy, childhood	Myopathy, lactic acidosis and sideroblastic anaemia (MLASA)
	*MTFMT*	AR	Infancy, childhood	Encephalomyopathy
	*TRIT1*	AR	Childhood	Encephalopathy, myoclonic epilepsy
tRNA synthetases	*AARS2*	AR	Infancy	Hypertrophic cardiomyopathy, myopathy
	*CARS2*	AR	Childhood	Epileptic encephalopathy, myoclonus
	*DARS2*	AR	Childhood, adulthood	Leukoencephalopathy in brainstem and spinal cord involvement and lactate elevation (LBSL)
	*EARS2*	AR	Infancy, childhood	Leukoencephalopathy with thalamus and brainstem involvement and high lactate (LTBL)
	*GARS*	AD	Childhood, adulthood	Charcot-Marie-Tooth 2D, Hereditary motor neuropathy 5A
	*MARS2*	AR	Childhood, adulthood	Autosomal recessive spastic ataxia and leukoencephalopathy (ARSAL) in French Canadians
	*RARS2*	AR	Infancy	Pontocerebellar hypoplasia type 6
	*SARS2*	AR	Infancy	Tubulopathy (hyperuricemia, metabolic alkalosis), pulmonary hypertension, and progressive renal failure (HUPRA)
	*YARS2*	AR	Childhood, adulthood	MLASA
Mitochondrial dynamic network (mitochondrial membrane biogenesis and maintenance)				
Fusion	*MFN2*	AD	Childhood, adulthood	Charcot-Marie-Tooth 2A (CMT2A) (multiple deletions)
	*OPA1*	AD	Childhood	Optic atrophy (multiple deletions)
	*OPA3*	AD	Adulthood	Optic atrophy
		AR	Infancy, childhood	Type III 3-methylglutaconic aciduria, Costeff syndrome
	*PINK1*	AR	Childhood, adulthood	Juvenile Parkinson Disease
Fission	*DNM1L* (*DRP1*)	AR	Infancy	Microcephaly, lactic acidosis, optic atrophy

*AR* autosomal recessive, *AD* autosomal dominant

The inheritance pattern of mitochondrial disease is dependent on the genetic mutations. Point mutations in the primary mitochondrial DNA such as m.3243A>G, three common LHON mutations, m.8344A>G, m.8993T>G/C, m.1555A>G and others are maternally inherited but sporadic mutations exist [53]. Single, large deletions in mtDNA are a common cause mitochondrial disease and they occur sporadically with rare exceptions [15]. The inheritance patterns of mutations in nuclear genes causing mitochondrial disease include autosomal recessive, dominant or X-linked. Both recessive and dominant forms exist in several mtDNA maintenance genes, for examples *POLG*, *PEO1* and *RRM2B* genes.

## Epidemiology of mitochondrial disease

The overall prevalence of mitochondrial disease is comparable to other neurogenetic diseases such as Charcot-Marie-Tooth (CMT) disease, myotonic dystrophy and muscular dystrophy. The prevalence of adult mitochondrial disease, both affected patients and those at risk, has recently been reported to be approximately 1 in 4300 in North East England [28]. Primary mutations in the mtDNA are more prevalent in the adult patients compared to mutations in the nuclear genes, and vice versa in the paediatric population where there is a much higher incidence of autosomal recessive disease particularly in consanguineous families. Although several hundreds of mutations have been reported in mtDNA since 1988, a handful of mutations are far more common than the others, for examples m.1555A>G (associated with aminoglycoside induced deafness), m.3243A>G (associated with MELAS syndrome and MIDD), m.3460G>A and m.11778A>G (associated with LHON), have an estimated prevalence of 0.19, 0.14, 0.11 and 0.11 %, respectively, in the population [11, 21]. However, individuals with these common mutations may remain clinically asymptomatic throughout their life if not exposed to relevant toxin or if they have a low mutation load.

## Clinical diagnosis

Mitochondria are ubiquitous and therefore mitochondrial disease can affect any organ, although organs with high energy demand such as brain, skeletal muscle and heart, are more commonly affected than the others. The clinical features are heterogeneous and often can mimic many neurological or other systemic diseases. Multi-system involvement is often evident with detailed clinical examination and investigations in most cases although there are exceptions such as Leber hereditary optic neuropathy (LHON). Paediatric onset disease is associated with more severe multi-systemic involvement, relentless progression and poorer prognosis, however, there are rare exceptions such as reversible respiratory chain deficiency caused by the m.14674T>C mutation [33].

Many classic syndromes have been described over the last few decades. The examples of clinical syndromes associated with adolescence and adulthood include mitochondrial encephalomyopathy, lactic acidosis with stroke-like episode (MELAS), myoclonic epilepsy with ragged red fibres (MERRF), mitochondrial neuro-gastrointestinal involvement and encephalopathy (MNGIE), neuropathy, ataxia and retinitis pigmentosa (NARP), chronic progressive external ophthalmoplegia (CPEO). In contrast, syndromes with neonatal and childhood onset include Alpers disease, Pearson syndrome, Leigh disease, Sengers syndrome and Kearns-Sayre syndrome.

However, clinicians are more commonly confronted by the non-specific constellation of clinical features. Many symptoms associated with mitochondrial disease such as deafness, diabetes, myopathy, gastrointestinal symptoms and others are also common on their own in the population but the ‘unusual’ combination of these symptoms in the same individual should alert the clinicians about the possibility of mitochondrial disease. Detailed system-based examination coupled with extended investigations is necessary to identify other system involvement such as short stature, sensori-neural deafness, pigmentary retinopathy, optic atrophy, diabetes mellitus and/or other endocrine dysfunction, cardiac involvement, renal tubulopathy and others. This systemic involvement may be subtle and asymptomatic in the early phase of disease. Disease rating scales have been utilised to document the extent of system involvement, disease burden and progression in paediatric and adult patients [62, 69].

Family history can be informative and often reveals what appear to be seemingly unrelated diseases among the maternal family members in primary mtDNA disease and the m.3243A>G mutation is a prime example [54]. It is important to ascertain history of consanguinity when recessive disease is suspected. Late adulthood presentation and/or lacking of apparent family history should not deter testing for mitochondrial disease because sporadic mutations or late presenting autosomal dominant diseases are not uncommon.

## Acute neurological presentations

### Stroke-like episodes and acute symptomatic seizure

MELAS syndrome is a severe, multi-system disease characterised by recurrent metabolic strokes with typical onset of below 40 years [31] although patients at the older age have been reported [6, 92]. Headache and prominent visual disturbance (both positive and negative symptoms) are often the prodrome of acute stroke-like episodes and can occur days or weeks before the development of focal neurological deficit or motor seizure. These visual symptoms can masquerade as migranous visual aura but in fact is the onset of occipital seizure [24, 35]. The severity of neurological deficits are related to the extent of parietal, temporal and occipital lobe involvement such as dysphasia, dyspraxia, heminanopia, cortical blindness, mild hemiparesis and psychosis. Epilepsia partialis continua and less commonly generalised status epilepticus occur during the stroke-like episodes in some patients.

**Fig. 1:**
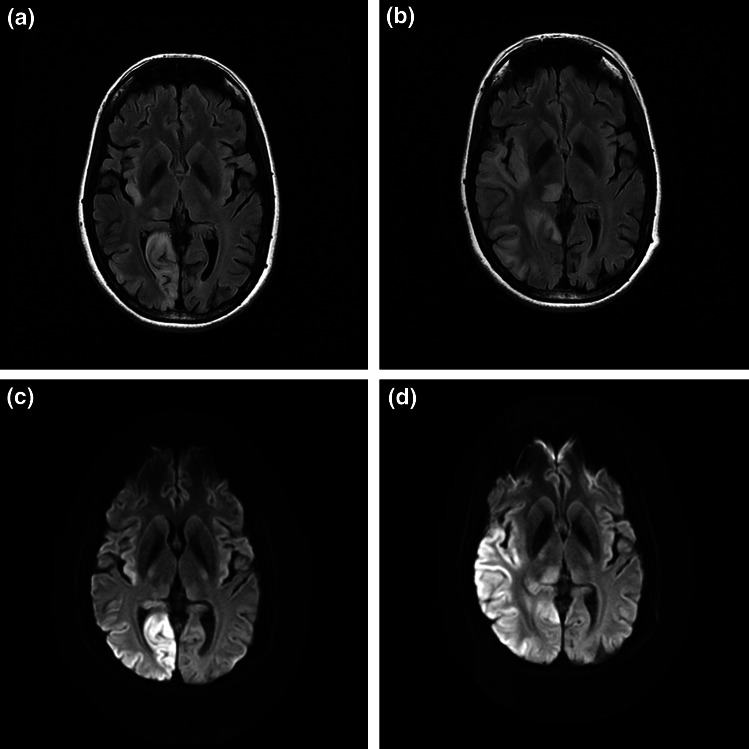
Axial FLAIR (**a**, **b**) and DWI (**c**, **d**) sequences of MRI head. **a** and **c** were performed on admission whilst **b** and **d** were performed 8 days later. The stroke-like lesion ‘spread’ from the right occipital lobe to the right temporal lobe and thalamus

The key imaging findings are cortical and subcortical lesions that cross the vascular territories of middle cerebral artery and posterior cerebral arteries and bilateral, asymmetrical changes are not infrequent (Fig. 1). The most common cause of MELAS phenotype is m.3243A>G mutation which accounts for 80 % of cases but mutations in the nuclear gene *POLG*, encoding for the catalytic subunit of DNA polymerase γ (pol γ), can cause similar stroke-like lesions [14, 19]. However, the *POLG*-related disease often has more aggressive disease course with explosive onset of focal seizure and status epilepticus that is highly refractory to pharmacological treatments in children and young adults and the outcome is very poor. Administration of sodium valproate is recognised to trigger fulminant hepatic failure among patients with *POLG* disease [73, 80]. In contrast, patients who have stroke-like episodes associated with the m.3243A>G mutation tend to have pre-existing, and often subtle, multi-system involvement and they often make good recovery in conjunction with the partial or complete resolution of imaging changes within few weeks or months at the early course of disease if appropriately managed (refer to Treatment section). Nevertheless, recurrence of stroke-like episodes leads to cumulative neuronal loss and results in severe cognitive impairment.

Given the high prevalence of m.3243A>G mutation and common carrier status of several pathogenic variants in *POLG* gene (p. A467T, p. W748S and p. G848S) in populations of European descent [29], clinicians should prioritise mitochondrial disease as a main differential diagnosis to atypical, evolving posterior circulation stroke, recurrent ‘encephalitis/encephalopathy’ with negative infective screen, auto-antibodies and ‘atypical/recurrent’ posterior reversible encephalopathy syndrome (PRES). Securing the diagnosis of mitochondrial disease early can avert the patients having invasive diagnostic procedure such as brain biopsy or potentially harmful treatment such as immune-suppressants.

### Subacute visual loss

The classic presentation of LHON is subacute, evolving painless visual loss in young adults with male predominance (4:1) [43]. Majority of the cases have the contralateral eye affected within a year and visual loss is often irreversible. Although there is a conventional belief that LHON mutations only affect eyes, other neurological features such as dystonia [48], myoclonus [41], sensori-neural deafness [66] may occur which broaden the spectrum of the clinical phenotype in these mutations [48]. It is clear that therefore LHON should be considered as a differential diagnosis to the ‘atypical’ optical neuritis that shows no recovery and Susac’s syndrome [97]. A link between LHON mutations and multiple sclerosis was speculated for a long time and a recent study has suggested that such association may occur by chance even though mechanistic interaction is possible [57].

Dominant optic neuropathy is caused by mutations in the nuclear gene such as *OPA1* and the irreversible visual loss often occurs in the childhood [95] although late-onset optic neuropathy after 4th decade [2] and adult-onset CPEO and parkinsonism with subclinical optic neuropathy have recently been described [12].

## Chronic neurological presentations

### Chronic progressive external ophthalmoplegia and ptosis

More than half of adult patients have external ophthalmoplegia and/or ptosis in our large cohort of patients (unpublished). Ptosis and ophthamoplegia can be asymmetrical at the outset but most cases become symmetrical with time. CPEO is one of the main presenting features in adult patients with mitochondrial disease [77] and ptosis often imposes more functional limitation than the restricted eye movement because the extra-ocular paresis occurs insidiously allowing cerebral adaptation and symptomatic diplopia is less common. Some patients with CPEO phenotype are occasionally misdiagnosed as other conditions such as seronegative myasthenia gravis.

### Myopathy

Many adult patients experience fatigue, exercise intolerance and muscle weakness. The degree of proximal myopathy is often mild on clinical testing and it progresses slowly. Early loss of ambulation due to muscle weakness is not a typical feature of adult mitochondrial disease with a few exceptions such as patients with Kearns–Sayre syndrome caused by single deletion in mtDNA and *TK2* mutation [4]. There is a risk of aspiration when facial and oropharyngeal weakness is present in addition to the respiratory muscle weakness. Some patients may have normal muscle strength and EMG study but complain of limited exercise capacity with recurrent nausea and/or vomiting on exertion due to lactic acidosis.

### Ataxia

Cerebellar ataxia is often subtle at the onset and typically progress with time and becomes debilitating in many genotypes. Cerebellar atrophy is a common imaging finding. Sensory ataxia due to dorsal root ganglionopathy is one of the defining features in SANDO phenotype (sensory ataxia, neuropathy, dysarthria and ophthalmoplegia) caused by *POLG* mutations [42].

### Neuropathy

Diminished or absent long tendon reflexes are a common clinical finding and axonal neuropathy is the most common finding in nerve conduction studies in adult patients with mitochondrial disease with a few exceptions such as demyelinating neuropathy described in patients with MNGIE [25]. A recent study showed that neuropathy is a useful feature to guide molecular diagnosis in adult patients with CPEO [32].

## Diagnostic approach

Comprehensive diagnostic criteria and guidelines have been published to provide a framework for clinicians when investigating patients with suspected mitochondrial disease [8, 85, 86]. Patients who present with classic syndromes such as MELAS, MERRF, LHON and Alpers disease can be diagnosed by direct sequencing of mitochondrial genes or *POLG* gene in blood. It is important to consider that blood heteroplasmy (leucocytes) declines with age in m.3243A>G [65] and ‘false negative result’ is possible in older adults therefore concomitant testing of additional tissue such as urinary epithelium is recommended. Some nuclear gene mutations have been found to have association with distinctive radiological appearances which can expedite the candidate gene sequencing, these include leukoencephalopathy with brainstem and spinal cord involvement and lactate elevation (LBSL) caused by *DARS2* mutations [89] and leukoencephalopathy with thalamus and brainstem involvement and high lactate (LTBL) caused by *EARS2* mutations [78].

However, many mitochondrial diseases do not have pathognomonic features that point towards a particular genetic diagnosis and muscle biopsy remains important in current clinical practice. The findings of muscle biopsy that are supportive of mitochondrial disease include: ragged red fibres (RRF), COX-negative fibres, individual complex or multiple respiratory chain deficiency, qualitative (multiple deletions) and quantitative (depletion) abnormalities in mtDNA.

Next generation sequencing is a new and high-throughput technique that allows sequencing of multiple candidate genes simultaneously leading to a more rapid diagnosis and increase the diagnostic yield [88] especially in the well-phenotyped cohort of patients [84]. Clinical exome sequencing is likely to become part of standard clinical care for undiagnosed patients.

## Treatment and long term management

### Specific treatment in mitochondrial disease

Currently, there remains no effective and specific treatment for vast majority of patients with mitochondrial disease. Various treatments (mostly nutritional supplements) such as co-enzyme Q10, carnitine, creatine, dichloroacetate and vitamin ‘cocktails’ have been widely used based upon anecdotal data and individual case reports, however, Cochrane systemic review of treatments that were tested in randomised-control trials concluded that none of these treatment showed meaningful clinical efficacy [59]. Since then, another randomised, controlled trial using idebenone in LHON mutations found no significant result in the primary end point defined as the best recovery in visual acuity but post hoc interaction analysis suggested benefits in those with discordant visual acuities [38]. EPI-743, a novel anti-oxidant has been reported to show clinical improvement in small number of patients with Leigh syndrome [47] and LHON [67] in the open-label clinical trials and phase 2B randomized-control trial is currently in progress.

Although there is no formal clinical trial, supplement of high dose co-enzyme Q10 (up to 2400 mg in three divided doses in adults; 30 mg/kg in paediatric cases) in primary co-enzyme Q10 biosynthetic defect has been reported to show variable clinical improvement across different phenotypes [23].

l-arginine has been reported to be effective in treating acute stroke-like episodes associated with m.3243A>G mutation [39]. However, this result is yet to be replicated by other research groups.

Allogenic haematopoietic stem cell transplant has emerged as a promising therapeutics to restore the enzymatic function in patients with MNGIE caused by *TYMP* mutations but this treatment is associated with high morbidity and mortality [25, 75].

### Supportive treatment and surveillance for complications

Symptomatic treatment and screening for associated complications remain fundamental to the management of mitochondrial disease.

#### Acute seizure and stroke-like episodes

Early recognition and prompt, aggressive seizure management are crucial to mitigate the cellular metabolic crisis perpetuated by the ictal activities [10]. The seizure management should follow the guidelines on status epilepticus except sodium valproate is absolutely contra-indicated in *POLG* mutations. Phenytoin was implicated in causing paralytic ileus in a patient with stroke-like episodes [17], however, this is likely to be a co-incidental finding as pseudo-obstruction is a common complication associated with MELAS syndrome [74] and we use this drug regularly to control seizures. Anecdotal evidence on the use of magnesium infusion [91], ketamine [64], ketogenic diet [34, 46, 79], folinic acid supplement [30] in termination of status epilepticus associated with *POLG* and m.3243A>G has been reported.

#### Pseudo-obstruction

Several genetic mutations (*TYMP*, m.3243A>G and *POLG*) have been associated with intestinal pseudo-obstruction [9, 63, 83, 90] involving small and/or large intestine. Distinguishing this from mechanical obstruction is of paramount importance as they tend to resolve with conservative management alone and surgery has little role and could exacerbate the metabolic crisis. Serum lactate is not a reliable marker for tissue ischaemia in patients with mitochondrial disease because some patients have persistently raised lactate even when they are well. Aggressive medical management of acute episodes is important as is prevention by using regular laxatives.

#### Cardiac involvement

Cardiac involvement is often part of the multi-system manifestation in adult mitochondrial DNA disease [7] although isolated cardiomyopathy has been reported in rare mtDNA mutations [26]. Cohort studies have shown that hypertrophic cardiomyopathy and pre-excitation syndrome are prevalent in m.3243A>G [37, 45] and m.8344A>G [13, 44] mutations, whereas conduction defect necessitating pacemaker is associated with single, large scale deletion particularly among those who have Kearns-Sayre syndrome. Currently, there is limited longitudinal data studying the prevalence of cardiac involvement in various nuclear genes in adults [60]. Baseline cardiac assessment with electrocardiogram (ECG) and echocardiogram should be performed in all patients and cardiac magnetic resonance in selected cases. Subsequent cardiac surveillance should be tailored according to the initial findings but the recommended interval is every 12 to 24 months [22, 55].

#### Diabetes mellitus

Diabetes mellitus is common in patients with m.3243A>G and single, large scale mtDNA deletions [70]. Metformin is best avoided because of the theoretical risk of lactic acidosis. Treatment of mitochondrial diabetes is otherwise similar to the usual form of diabetes although it appears that it is associated with a more rapid progression to insulin therapy [93].

#### Ptosis

A proportion of patients with CPEO develop significant ptosis that obscures visual field. Corrective ptosis surgery such as frontalis sling operation improves the functional and cosmetic outcome in selected patients [3].

#### Deafness

Young onset, bilateral sensori-neural deafness is prevalent in mitochondrial disease. The quality of life of many patients can simply be improved with digital hearing aid and cochlear implant can be reserved for those with severe hearing loss [36, 81].

## Genetic counselling and reproductive options

Similarly to other genetic disorders, screening for family members at risk and offering genetic counselling is essential in mitochondrial disease. For patients with nuclear gene disorders, genetic counselling and reproductive options are identical to other nuclear defects. For women with mtDNA mutations genetic counselling is a challenging area. Patients should be reassured if they harbour sporadic mutation, such as single, large scale mtDNA deletion, because risks of transmission are low. For mtDNA point mutations, accurate elucidation of the risk of transmission and prediction of disease status is extremely challenging due to the genetic bottleneck effect and uneven tissue segregation associated with some point mutations. It is estimated that there are approximately 152 births per year in the UK of children who carry potentially pathogenic mitochondrial DNA mutations [27]. In view of the complexity of mtDNA genetics, referral of child-bearing age female patients to specialist centres for discussion of reproductive options is recommended. The available options are chorionic villous sampling (CVS), amniocentesis [52] and preimplantation genetic diagnosis (PGD) [68, 87]. CVS and amniocentensis are performed at different stages of pregnancy, 10–12 and 14–20 weeks, respectively. PGD is an IVF procedure that involves embryo biopsy and the selection of embryos with the lowest mutation load. However, PGD will not benefit carriers with homoplasmic mtDNA mutation.

Mitochondrial donation, either pronuclear transfer [18] or metaphase II spindle transfer [82], is emerging as a potential reproductive option to prevent the transmission of mtDNA mutations. In the UK after many years of debate and scientific scrutiny, Mitochondrial Donation Regulations, were passed by both Houses of Parliament, making mitochondrial donation legal for the first time in the UK. The Human Fertilisation and Embryology Authority will now develop a licencing framework through which applications can be considered on a case by case basis.

## Conclusions

Over recent years there have been important advances in mitochondrial disease, particularly in terms of diagnosis and reproductive options available. The role of the clinician remains crucial since a high index of clinical suspicion and prompt recognition of complications remain essential to make an earlier diagnosis and instigate a better management. Currently, the management of mitochondrial disease is largely supportive; however, with the improved understanding of disease mechanisms, ongoing treatment trials and discovery of new therapeutic agents should give hope for patients with mitochondrial disease.
